# The adaptive immune response to cardiac injury—the true roadblock to effective regenerative therapies?

**DOI:** 10.1038/s41536-017-0022-3

**Published:** 2017-06-19

**Authors:** Susanne Sattler, Paul Fairchild, Fiona M. Watt, Nadia Rosenthal, Sian E. Harding

**Affiliations:** 1 National Heart and Lung Institute, Imperial College London, London, W12 0NN UK; 2 Sir William Dunn School of Pathology, University of Oxford, Oxford, OX1 3RE UK; 3 Centre for Stem Cells & Regenerative Medicine, King’s College London, London, SE1 9RT UK; 4 The Jackson Laboratory, Bar Harbor, ME 04609 USA

## Abstract

The regenerative capacity of adult human tissues and organs is limited, but recent developments have seen the advent of promising new technologies for regenerative therapy. The human heart is of particular interest for regenerative medicine, as cardiac tissue damage is repaired by the formation of rigid scar tissue, which causes inevitable structural changes and progressive functional decline leading to heart failure. Cardiac regenerative medicine aims to prevent scar formation or replace existing scars to halt or reverse adverse remodeling and therapeutic approaches include the use of biomaterials, gene therapies, delivery of growth factors, and (stem) cell therapies. Regenerative therapies, however, face significant obstacles in a hostile microenvironment. While the early immune response to a myocardial infarct is essential to ensure tissue integrity and to avoid fatal cardiac rupture, excessive activation of endogenous repair mechanisms may lead to ongoing inflammation, fibrosis, and sustained autoimmune-mediated tissue damage. Anti-cardiac autoreactivity of the adaptive immune system has been suggested to be involved in structural remodeling, functional decline, and the development of heart failure. It is, therefore, crucial to first understand the endogenous response to cardiac tissue damage and how to restore immune tolerance to cardiac tissue, before additional regenerative therapies can achieve their full potential.

## Introduction

Heart failure after an acute myocardial infarct (MI) remains a significant cause of mortality and a major clinical challenge. An infarct induces severe tissue damage and the immune system is crucial in orchestrating the various steps of the post MI healing process. A tightly controlled interplay between immune cell populations is responsible for removing cellular debris, restoring tissue integrity to prevent fatal cardiac rupture, and then quickly resolve inflammation. An appropriate balance between inflammatory and regenerative mechanisms is essential, as excessive early inflammation may cause collateral damage to healthy tissue and is detrimental to healing. While beneficial vs. detrimental effects of early innate immune populations, including neutrophils and macrophages, have received considerable attention in recent years,^1^ the role of the adaptive immune system at later stages and during cardiac remodeling is far from understood. Importantly, post MI patients present elevated levels of anti-cardiac auto-antibodies,^2^ consistent with the involvement of the adaptive immune system and indicating an aberrant activation by self-antigens causing anti-cardiac autoimmunity. The notion that the development and progression of heart failure is promoted by an underlying autoimmune-inflammatory condition, maintaining persistent low levels of tissue damage that sustain cardiomyocyte loss, fibrosis, and pathological ventricular remodeling is recently gaining support.

We propose that restoring cardiac immune tolerance by immunomodulatory interventions post MI is essential to pave the way for more efficient outcomes of regenerative therapies.

## MI and heart failure

Recent advances in the clinical response to MI have significantly reduced acute mortality.^3^ However, endogenous repair mechanisms gradually replace necrotic cardiac tissue by a non-contractile scar, which may lead to ventricular remodeling. This is due to the overstretching of surviving cardiomyocytes to maintain cardiac output, leading to thinning of the ventricular wall and development of heart failure.^4, 5^ Current management of heart failure relies on beta-blockers, diuretics, and vasodilators such as angiotensin converting enzyme inhibitors. Surgical options are to change the shape of the left ventricle or implant assist devices such as pacemakers or defibrillators.^6^ However, these treatments do not completely stop progression of disease and cannot restore heart function, presenting an urgent clinical need for curative treatment options that target underlying mechanisms. Current experimental approaches to treat heart failure largely focus on cardiomyocyte deficiency and aim to replace lost cardiomyocytes in the infarcted area. Efforts to increase cardiomyocyte numbers target the limited proliferative capacity of pre-existing cardiomyocytes or aim to differentiate endogenous or delivered progenitor cells into cardiomyocytes.^7^ Recent advances have been reviewed extensively^8, 9^ and include (1) biomaterial-based approaches,^10^ (2) gene therapy using plasmid DNA for gene transfer^11^ or silencing/short hairpin RNA, microRNA, and long non-coding RNA for gene modulation,^12, 13^ (3) exogenous administration of growth factors such as neuregulin^14^ or fibroblast growth factor,^15^ and (4) cell therapies using mesenchymal stromal cells (MSCs),^16–18^ cardiac and bone marrow-derived c-kit^+^ cells,^19, 20^ cardiosphere cells,^21, 22^ and pluripotent stem cell-derived cardiac cells.^23^ While promising results have been obtained in experimental pre-clinical models, many of these approaches still face severe obstacles concerning efficacy of production and delivery, as well as immunological rejection. In particular though, they all share the common hurdle of a hostile inflammatory microenvironment and so far benefits observed in clinical trials are disappointing or inconsistent.^9, 23^

## Post MI autoimmunity

The immune system is a sophisticated system of cells and effector molecules essential to protect the host from pathogens and ensure tissue integrity. Two fundamental features to ensure these roles are the ability to discriminate between the body’s own (self) and foreign (non-self) agents and to react quickly if there are signs of “danger” usually associated with tissue damage, including the release of intracellular molecules (danger-associated molecular patterns, DAMPs) such as high-mobility group box-1, heat shock proteins, nucleic acids, and mitochondrial molecules.^24^ Tight regulatory networks are in place to restrain immune responses to self-antigens and to downregulate a response once an initial insult is cleared to minimize inflammatory damage. If these regulatory networks fail, chronic inflammatory conditions may develop, including allergies and autoimmunity. Autoimmune disease is a highly diverse group of conditions, classified according to main target organs and manifestations. While a certain level of immunological autoreactivity is normal and generally kept in check, an environmental trigger in a genetically susceptible individual can overwhelm regulatory mechanisms and result in immune-mediated tissue destruction.

**Fig. 1:**
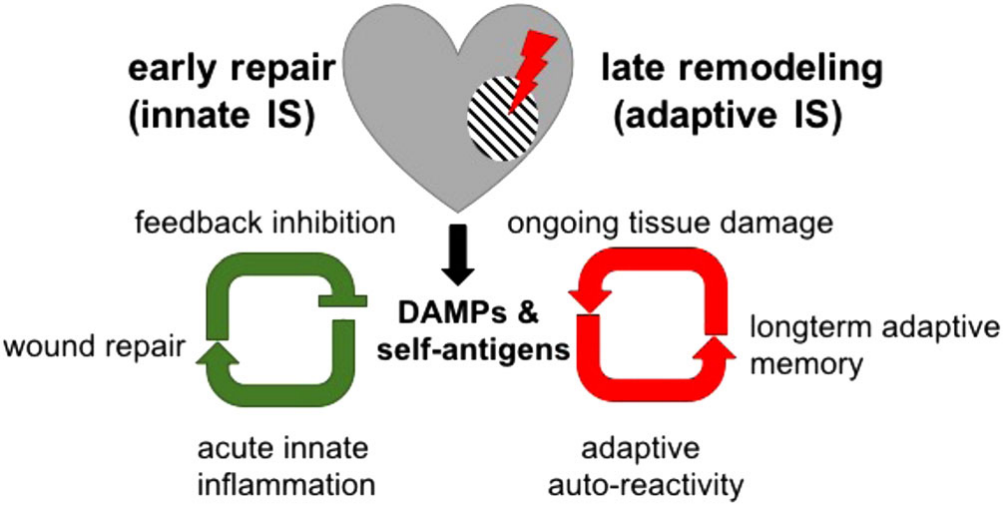
The role of the innate and adaptive immune system post MI. A MI causes severe tissue damage and the release of DAMPs and cardiac self-antigens such as myosin and troponin. Immediately after injury, DAMPs lead to an acute inflammatory response, characterized by the influx of a vast number of innate immune cells, which initiate and orchestrate wound repair. Negative feedback mechanisms activated immediately and the declining availability of DAMPs eventually resolves early inflammation. However, in parallel, the release of massive amounts of self-antigens in an inflammatory environment breaks tolerance mechanisms and induces long-lived antigen-specific adaptive immune cells, which cause ongoing autoimmune tissue damage and continuous supply of self-antigen. IS; immune system

Physical trauma and the associated release of DAMPs from dying cells causes local and systemic inflammation and can be a potent trigger for the induction of autoimmunity.^25^ The release of previously sequestered antigens from necrotic cells progressively diversifies the autoreactive lymphocyte repertoire in a process termed epitope spreading, which is well established as a crucial contributor to chronic autoimmune tissue destruction.^26^ An infarct causes severe tissue trauma, with necrotic heart tissue releasing vast amounts of DAMPs together with cardiac proteins. In these highly inflammatory circumstances, the ectopically encountered cardiac antigens are recognized by autoreactive lymphocyte clones and can induce autoimmunity. These antigens include α-myosin heavy chain, a prominent example of an abundant cardiac protein that is commonly targeted by post MI anti-cardiac autoreactivity.^27^ Subsequent immune-mediated tissue injury provides an ongoing supply of the same auto-antigens, culminating in persistent immune autoreactivity (Fig. 1).

## Auto-antibodies and B-lymphocytes

An abundance of clinical literature, spanning over three decades, documents the presence of auto-antibodies specific to cardiac antigens in patients with a wide range of heart diseases.^2, 28^ Antibody-mediated cardiac damage results from binding to and influencing signaling of surface receptors including β-adrenergic and muscarinic receptors^29, 30^ or from immune complex-mediated direct cellular toxicity.^31^ However, assigning a causative pathogenic role to specific anti-cardiac antibodies is technically challenging and most reports rely on their presence and theoretical ability to cause damage. Importantly, low titers of auto-antibodies to various antigens can also be detected in healthy subjects, where they do not cause any clinically evident pathology.^32^ A lack of inflammatory stimulation presumably allows control mechanisms to work appropriately, and under physiological conditions many intracellularly localized cardiac antigens such as myosin and troponin are not easily accessible to the immune system.

Information on other B-lymphocyte functions in MI, such as the secretion of cytokines and growth factors, and antigen presentation, is missing to date. B-lymphocytes infiltrate the mouse myocardium after experimental MI^33^ and one B-lymphocyte depletion study shows improved post MI recovery of heart function.^34^ Moreover, activated B-lymphocytes produce cytokines depending on their environment^35^ and excessive levels of pro-inflammatory cytokines such as TNF-α directly contribute to myocardial dysfunction by depressing contractility, inducing myocyte apoptosis,^36^ and inducing fibroblast differentiation into myofibroblasts, a crucial step toward myocardial remodeling.^37^

## T-lymphocytes

The involvement of T-lymphocytes in post MI repair and regeneration has been investigated only recently. However, the presence of mature B-lymphocytes producing isotype-switched antibodies, which are dependent on antigen-specific T helper cells, has long provided indirect proof of their involvement. Knowledge about the exact role of T-lymphocytes post MI is still very limited, but considering the diversity of the T-lymphocyte population and the rapidly changing environmental conditions in the heart and the periphery after an infarct, it is likely that there will be tightly regulated changes in subpopulations, effector functions, and beneficial or detrimental effects depending on time point and exact type of damage. Results to date show that post MI effector T-lymphocytes are antigen-specific, primed in the heart-draining lymph nodes and infiltrate the heart.^33, 38^ Their infiltration of the tissue after activation in the lymph node suggests roles beyond B-lymphocyte activation, but corresponding data are scarce and highly dependent on model system and exact analytical timepoint. Immediately post MI, regulatory T-lymphocytes appear to play a beneficial role.^39, 40^ Conversely, CD4^+^ effector T-lymphocytes produce inflammatory cytokines such as IFN-γ and IL-17 in the post MI myocardium^38^ and pro-inflammatory cytokines are known to increase cardiomyocyte death and enhance fibroblast proliferation and pro-fibrotic gene expression.^41^ Direct cytotoxic effects of infiltrating CD8^+^ T-lymphocytes have also been suggested.^42^ In experimental autoimmune myocarditis, CD4^+^ helper T-lymphocytes producing IFN-γ and autoreactive cardiac myosin-specific cytotoxic CD8^+^ T-lymphocytes are major mediators of cardiac damage,^43, 44^ which suggests similar roles for these two major broad T-lymphocyte subpopulations in post MI cardiac autoreactivity.

## Dendritic cells (DCs)

Although not traditionally viewed as part of the adaptive immune system, DCs are crucial in the activation of adaptive antigen-specific T-lymphocytes. Through their activation state when presenting antigen, they direct T-lymphocytes toward either an effector or regulatory phenotype. Under homeostatic conditions, presentation of self-antigen induces a regulatory/tolerogenic phenotype, while in an activated context inflammation-mediated activation of DC and antigen presentation may induce autoreactive effector lymphocytes. We and others have demonstrated that DC numbers indeed increase in infarcted hearts in experimental models^45^ and decreased numbers of DC in human infarcted myocardial tissue are associated with impaired reparative fibrosis and the development of cardiac rupture soon post MI.^46^ This early benefit and role in fibrotic repair, however, may well present a threat at later stages, causing excessive fibrosis and subsequent remodeling. The role of endogenous DC populations in inducing and shaping post MI cardiac autoreactivity and their impact on cardiac remodeling toward heart failure is currently under investigation.

## How does adaptive autoreactivity impede regenerative therapies?

It is accepted that impediments to therapy exist immediately after an infarct, when the innate immune system responds with astounding speed and force. However, after the initial clearance of acute post MI inflammation, adaptive autoreactivity may proceed insidiously with potentially grave effects. An autoimmune response against major cardiac proteins is very likely to confound a full regenerative outcome in heart disease: it may even destroy tissue in previously unaffected regions of the heart and increase the strain on remaining healthy cardiomyocytes to compensate for the loss. This will exacerbate the remodeling process and accelerate maladaptive left ventricular dilation and heart failure.

Regenerative therapies therefore not only have to deal with the status quo of damage at the time of administration, but also face ongoing autoimmune tissue destruction. Some therapies rely on local delivery of cells and factors into the infarct zone; an approach that seems most efficient considering retention of therapeutic agents where they seem to be needed most, but fails to address ongoing remote damage.

In addition, regenerative therapies, particularly those involving allogeneic cells, must withstand an immunologically distorted environment both in the periphery and in the local microenvironment of the tissue.^47^ Increased levels of inflammatory cytokines may aggravate immune rejection and prevent engraftment, or even change therapeutic cell phenotypes and impair appropriate function of exogenously delivered stem cells.^48^

## From immunosuppression to immunomodulation

The scenarios described above may give the impression that the immune system in its entirety is adverse to efficient post MI healing and regeneration. On the contrary, it is fundamental in orchestrating the early healing response. Attempts to improve post MI cardiac regeneration by broad immunosuppression have resulted in poor healing, scar formation, and heart failure in animal models and clinical studies. General immunosuppression with corticosteroids and non-steroidal anti-inflammatory drugs disrupts the healing process by impairing collagen deposition and scar formation, leading to an increased chance of left ventricle rupture.^49–52^ Similar to full blown immunosuppression, clinical trials using intravenous immunoglobulin, which broadly blocks the full antibody repertoire as well as several other innate and adaptive immune pathways, have yielded limited and inconsistent benefits,^53, 54^ likely because they do not target the balance between regulatory/regenerative and inflammatory subpopulations of lymphocytes.

**Fig. 2:**
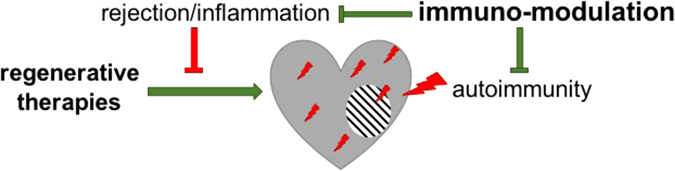
Regenerative therapies are hampered by direct and indirect immunological mechanisms. Immunological rejection and a hostile inflammatory environment may prevent stem cell engraftment or change their beneficial properties. At the same time, anti-cardiac autoreactivity causes further and additional tissue damage. Immunomodulatory therapies may prevent anti-cardiac autoimmunity as well as immunological rejection and excessive inflammation

Thus, the way forward is immunomodulation, aiming to restore cardiac tolerance and achieve appropriate balance between inflammatory and regulatory immune cell populations and factors (Fig. 2).^55^ Combination therapies of regenerative and immunomodulatory treatments have so far largely focused on preventing immune-mediated rejection of transplanted cells or materials by blocking of pro-inflammatory pathways or co-delivery of regulatory immune cells.^56^ However, similar principles could be applied to the restoration of endogenous cardiac tolerance. Notably, beneficial effects of some post MI therapies have in fact been attributed to a modulating effect on the immune response. For example, statins are in routine clinical use in cardiovascular disease and after an acute MI, with the primary goal of reducing cholesterol synthesis. However, statins are now known to modulate the immune response by enhancing regulatory T-lymphocyte numbers and function while inhibiting pro-inflammatory T-lymphocyte subpopulations.^57^ A prominent example of immunomodulatory cell therapy is the use of MSCs, which in themselves combine cell therapy with immunomodulation due to their stem cell-like and immunosuppressive characteristics.^58^

There are a variety of approaches to boost immune-regulatory mechanisms, but due to their central and unique role in defining the T-lymphocyte phenotype, DC are likely to be an ideal intervention point to skew the post MI anti-cardiac lymphocyte population toward tolerance. Notably, immunomodulatory cell therapies using DC have shown promising results in experimental pre-clinical settings as well as in clinical trials in autoimmune patients with type 1 diabetes or rheumatoid arthritis.^59, 60^ Indeed, induction of antigen-specific immune tolerance to cardiac antigens has been achieved in a rat model of cardiac damage, which resulted in a milder inflammatory infiltrate, less collagen deposition, and improved cardiac performance.^61^ Most relevant however, two very recent studies reported beneficial effects of cardiomyocyte antigen-specific tolerogenic DC on post MI function and remodeling in mice.^62, 63^ Importantly, priming DC with post MI serum was as protective as using cardiac tissue lysate, providing a first proof of concept that post MI tolerogenic DC therapy may be translatable into clinical use.^63^ Due to their migratory ability and systemic mode of action, DC can be injected subcutaneously, which will avoid common efficacy problems associated with intravenous or intramyocardial cell delivery. Tolerogenic DC can be generated from a patient’s own blood and treatment could be performed within a few days after infarct together with or preferably even before other regenerative therapies. It is therefore both crucial and feasible to design therapeutic strategies for cardiac regeneration targeting ongoing anti-cardiac autoreactivity.

## Open questions

Research aimed at unraveling the vast complexity of the endogenous immune response to injury is making rapid progress, but many unanswered questions remain.

### How is post MI cardiac autoreactivity induced and which immune cell types are involved at what stage?

Answers to this questions would allow specific targeting of detrimental vs. beneficial cell types, effector molecules or molecular pathways at the most effective window in time. Identification of the targeted auto-antigens might even allow for “counter-vaccination” to induce immune tolerance.

### What is the immunological role of cardiac resident cell types in exacerbating post MI immune responses and autoreactivity?

Cardiac cells including cardiomyocytes, endothelial cells, pericytes, fibroblasts, and tissue-resident macrophages are far from mere victims of an immunological attack but crucial active players in shaping the post MI immune response. A better understanding of their immunological characteristics and the cross-talk between cardiac cells and immunological factors may help to protect them from immune-mediated damage.

### What is the contribution of genetic diversity?

The exact immune response and the balance between cell populations and factors varies dramatically between individuals. Autoimmune prone (and known autoimmune) patients have a worse outcome post MI. Knowing which factors predispose a post MI patient to detrimental autoreactivity and rapid progression to heart failure may allow earlier and more successful intervention.

## Conclusion

Although the field is still in its infancy, there is accumulating evidence that cardiac regeneration is hampered by excessive activation of the immune system and that the progression of ischemic damage to adverse remodeling and heart failure in particular is exacerbated by an ongoing adaptive immune response. Current experimental therapies rely on the assumption that the damaging incident—the infarct—has passed, and it is now time to start repairing the myocardium. Unfortunately, this might be a flawed assumption. While acute early inflammation may be resolved, adaptive autoreactivity will linger, may cause persistent low-level tissue damage, even in previously healthy areas, and may counteract any beneficial effects of regenerative therapies.

The ideal post MI treatment to prevent progression to heart failure should therefore aim to induce immune tolerance to cardiac antigens as a priority. This will prevent the development of pathological adaptive immune autoreactivity, and thus provide other therapies with an environment truly permissive of regeneration.

### Data availability

Data sharing not applicable to this article, as no data sets were generated or analyzed during the current study.
